# A hidden problem: peripheral artery disease in women

**DOI:** 10.1093/ehjqcco/qcad011

**Published:** 2023-03-08

**Authors:** Mary M Kavurma, Lauren Boccanfuso, Carina Cutmore, Freda Passam, Sanjay Patel, Annemarie Hennessy, Jacky Loa, Gemma A Figtree, Jonathan Golledge, David A Robinson, Sarah Aitken

**Affiliations:** Heart Research Institute, The University of Sydney, Sydney, NSW, Australia; Heart Research Institute, The University of Sydney, Sydney, NSW, Australia; Faculty of Medicine and Health, The University of Sydney, Sydney, NSW, Australia; Concord Institute of Academic Surgery, Concord Hospital, Sydney, NSW, Australia; Heart Research Institute, The University of Sydney, Sydney, NSW, Australia; Central Clinical School, Faculty of Medicine and Health, The University of Sydney, Sydney, NSW, Australia; Heart Research Institute, The University of Sydney, Sydney, NSW, Australia; Department of Cardiology, Royal Prince Alfred Hospital, Sydney, NSW, Australia; Heart Research Institute, The University of Sydney, Sydney, NSW, Australia; School of Medicine, Western Sydney University, Sydney, Australia; Department of Vascular Surgery, Royal Prince Alfred Hospital, Sydney, NSW, Australia; Faculty of Medicine and Health, The University of Sydney, Sydney, NSW, Australia; Kolling Institute of Medical Research, Royal North Shore Hospital, Sydney, NSW, Australia; Queensland Research Centre for Peripheral Vascular Disease, College of Medicine and Dentistry, James Cook University, Townsville, QLD, Australia; The Department of Vascular and Endovascular Surgery, The Townsville University Hospital, Townsville, QLD, Australia; Department of Cardiology, Royal Prince Alfred Hospital, Sydney, NSW, Australia; Faculty of Medicine and Health, The University of Sydney, Sydney, NSW, Australia; Concord Institute of Academic Surgery, Concord Hospital, Sydney, NSW, Australia

**Keywords:** Peripheral artery disease, Gender, Socioeconomic pathways

## Abstract

Peripheral artery disease (PAD) has a huge social and economic burden and is an important contributor to the global health burden. Sex differences in PAD are apparent, with recent data suggesting equal if not greater prevalence in women, and women having worse clinical outcomes. Why this occurs is not clear. To identify underlying reasons for gender inequalities in PAD, we executed a deeper exploration through a social constructive perspective. A scoping review was conducted using the World Health Organization model for analysis of gender-related needs in healthcare. Complex interacting factors, including biological, clinical, and societal variables, were reviewed to highlight gender-related inequities in the diagnosis, treatment, and management of PAD. Current gaps in knowledge were identified and insights into future directions aimed at improving these inequalities were discussed. Our findings highlight the multi-level complexities that need to be considered for strategies to improve gender-related needs in PAD healthcare.

## Graphical Abstract

**Graphical Abstract:**
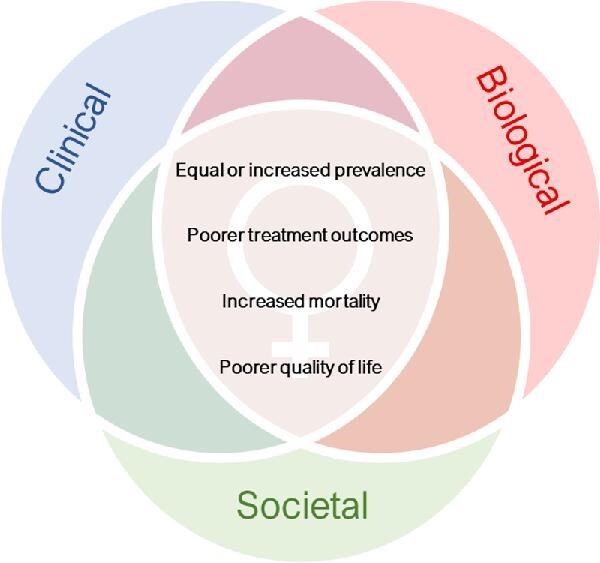
Biological, clinical and societal interactions that impact health-related inequalities in women with peripheral artery disease (PAD).

## Introduction

Peripheral artery disease (PAD) is the leading cause of lower limb amputation and a major risk factor for cardiovascular mortality. PAD affects >200 million people worldwide, and its prevalence expected to rise ∼50% by 2045.^1^ Increasingly, research is oriented towards understanding the health of under-represented populations, including gender-related inequalities. To design and implement effective and inclusive strategies aimed at improving person-centred care and health outcomes, it is necessary to explore the influence of sex and gender in PAD. In 2012, the American Heart Association (AHA) made a call-to-action to address gender-related disparities in PAD, noting the need to raise clinical awareness, focused treatment plans and expand research efforts.^2^ More than 10 years later, PAD is still underdiagnosed and understudied in women despite recent findings suggesting an increased prevalence in women.^2,3^

**Figure 1:**
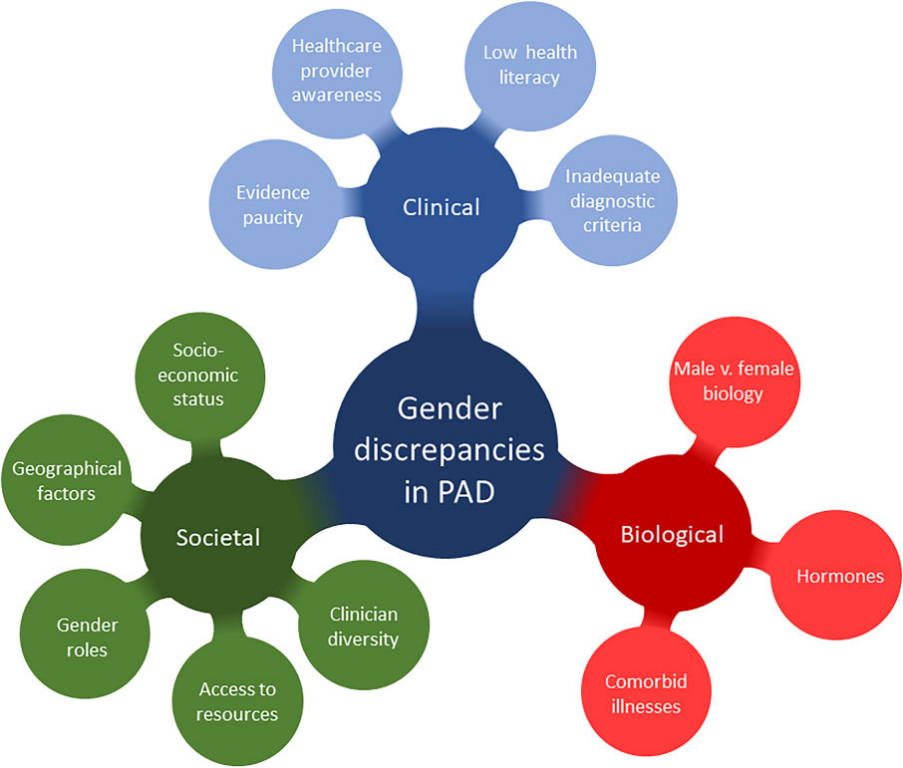
Gender discrepancies in peripheral artery disease (PAD).

To understand gender-related health inequalities, it is important to reach beyond traditional biological drivers of sex differences and consider socially constructed gender roles and relationships, inclusive of gender diverse people.^4^ Sex and gender interact in complex and multifaceted ways to influence health outcomes. Biological differences between men and women are also influenced by the gendered structures of health systems (access to and control of resources, aptitudes, and skills) and socioeconomic structures (gender roles and values in society). Research into intersectionality demonstrates how differences in age, culture, race, sexuality, and class mediate the influence of these factors. The World Health Organization (WHO) proposes a model for analysis of gender-related needs in healthcare (*Figure 1*).^5^ The WHO framework requires researchers evaluating gender inequalities to consider potential biological, clinical, and social mechanisms arising at different levels, from the microlevel variables (such as biology, individual behaviours, and risk factors), the mesolevel variables (including education, family, and employment), and macrolevel variables (such as the role of women in society, and healthcare systems). This approach also requires researchers to consider the intersection between the problem and systems both locally and globally.^6^ To meaningfully address gender inequalities in PAD, complex multilevel strategies are needed across all three contextual levels.

This review evaluates what is currently known about men and women with lower extremity PAD through a social constructivist perspective, to identify known contributors to gender-related inequalities in the diagnosis, treatment, and management of PAD. This methodological approach is unique in PAD research, as it places a stronger emphasis on the social determinants of health and PAD, considering the role of women in society, and provides a useful framework for understanding the influence of sex and gender on healthcare needs.

## Methodology

We conducted a comprehensive scoping review using MEDLINE, EMBASE, and SCOPUS, starting with key words: ‘peripheral artery disease’, ‘gender’, ‘sex’, ‘peripheral vascular intervention’. Consistent with the broad research questions in scoping reviews, the search was iterative, identifying new search terms with subsequent search rounds to reveal the depth and breadth of research on gender inequalities in PAD. We engaged in snowball searching using reference reviews to identify additional publications from selected studies. Data were analysed to describe themes related to gender inequalities, grouping results into relevant topic areas as determined by the WHO framework, supported by key references.

The authorship team collaboratively developed the study plan, drawing on our interdisciplinary expertise as scientists and clinicians (biomedical science, vascular surgery, and cardiology), which prompted critical reflection and diversity in analysis and interpretation, and supported the rigour of the review methodology. The primary review team engaged in reflexive discussion about how their intersectional identities (including gender, various culturally and linguistically diverse backgrounds, and professional backgrounds) influenced the research process at each stage. The social constructivist WHO framework used throughout this review helps synthesize the current evidence on gender disparities in PAD and substantially expands on previous review articles^7–9^ to address deeper complexities contributing to inequality, including the social determinates of disease.

## Gender inequalities in current PAD diagnostic and treatment paradigms

### Prevalence of PAD

Historical studies reporting at the incidence of intermittent claudication (IC) contributed to the idea that PAD is predominantly a disease affecting men.^10^ More recent epidemiological studies report women to have at least a similar, if not higher, prevalence of PAD to men, including women from low and middle-income countries (LMIC) and in socioeconomically disadvantaged groups.^2,3^ In 2019, Song and colleagues reported a higher prevalence for PAD from women >25 years of age in high-income countries with the prevalence equalizing by 65 years of age.^3^ These figures may in fact be an underestimate, since women are often asymptomatic or have atypical symptoms compared with men, making diagnosis difficult.^9^

### Clinical presentation and symptoms

*Table 1* summarizes diagnostic and treatment inequalities in women with PAD. PAD is traditionally classified into three clinical phases: asymptomatic, IC, and critical limb ischaemia—using systems such as the Fontaine^11^ or Rutherford^12^ scores; noting that the term critical limb ischaemia (referring to patients with rest pain, ulceration, or necrosis) was recently replaced by chronic limb-threatening ischaemia (CLTI).^13^ PAD does not always progress through all clinical stages, and these scoring systems are increasingly recognized as poorly representative of many patients’ symptoms of PAD,^13^ especially in women.^14^ Women have a lower prevalence of IC when compared with men,^14^ they also have ∼2-fold prevalence of CLTI,^15^ and more multi-level arterial occlusive disease.^16^ Furthermore, the typical presentation (IC) in women generally occurs much later in age than men, ∼10–20 years later, and post-menopause.^9^

**Table 1:** Diagnostic and treatment inequalities in women with peripheral artery disease (PAD)

Prevalence	• Generally higher prevalence equalizing after menopause.^2,3^
Clinical presentation and symptoms	• Atypical or absent symptoms.^14^ • Lower rates of intermittent claudication.^14,15,99^• Greater chronic limb-threatening ischemia as first manifestation of PAD.^14,15^• Multilevel disease.^16,35^• Aging.^9^
Diagnosis	• Ankle-brachial index measurements, but these are less sensitive with asymptomatic disease.^14,20^
Initiating therapy	• Lower rates of guideline-directed medical therapy adherence for PAD.^21^• Less likely to receive guideline-directed therapy.^22,23^• Lower rates of surgical intervention.^30,31^• Higher rates of endovascular treatment vs. amputation or open surgery^32^; more complications.^35–37^
Responses to therapy	• Reduced or no improvement with supervised exercise therapy.^26–29^• Higher mortality following amputation or open surgery.^35,37^

### Diagnosis of PAD

Most population-based screening studies for PAD use a reduced ankle-brachial index (ABI) of 0.9 to identify PAD. In women with reduced ABI, the prevalence of typical symptoms is less than that seen in men, with women more likely to be asymptomatic.^14^ These findings may represent differences in PAD symptom manifestation, or that the exertional leg pain in women may be attributed to other conditions.^17^

### Treatment

The major goal in the treatment of PAD is to manage symptoms to maintain quality of life, decrease major adverse limb events (ulceration and amputation), and minimize the risk of myocardial infarction (MI) and stroke. Clinical guidelines recommend lifestyle, pharmacological, exercise, and surgical treatments. Considerable inequalities in treatment exist between sexes.

#### Pharmacotherapy

Medical therapy and secondary risk prevention for PAD include statins, antiplatelets, antihypertensives, control of diabetes, and cessation of smoking.^13,18–20^ Criqui and colleagues recently reported a lack of guideline adherence, with only ∼11–67% adherence observed for the use of evidence-based preventative therapies in PAD.^21^ Women and older individuals have even lower rates of guideline-directed therapy,^22,23^ and when given, the impact of therapies may be different with the sexes, e.g. antithrombotic therapies,^24,25^ as described later.

#### Exercise

Supervised exercise training improves walking distance, reduces leg pain, and improves quality of life and is therefore recommended as first-line therapy for PAD. Women appear to have less improvement in walking distance after exercise programmes than men,^26–28^ while other reports show no sex-dependent associations after exercise.^29^

#### Surgical intervention

Open or endovascular techniques are recommended for patients with lifestyle-limiting symptoms unresponsive to exercise therapy or with CLTI.^20^ Less women proceed to surgical intervention for PAD,^30,31^ and when admitted to hospital for acute management, women often have an endovascular procedure rather than an amputation or bypass surgery compared with men.^32^ This may reflect a selection bias since females have higher mortality rates following amputation or open surgery.^33,34^ Women also have a greater in-hospital complications after endovascular surgery, including higher rates of bleeding, vessel access site complications, haematoma, or pseudoaneurysm.^35–37^ Following endovascular intervention, women also have a higher risk of dissection, amputation, MI, and death.^35,37^ Intersecting multimorbidity, particularly frailty, disproportionally affects women more than men, and leads to increased complications after endovascular and open surgery.^38,39^ This may, in part, be related to the smaller vessel size in females.^40^ Women also present with more complex lesions and comorbidities than men,^35^ as well as greater pain intensity in daily activities following limb loss.^41^ Conversely, the EUCLID trial reported women with symptomatic PAD (ABI ≤ 0.8) to be protected from major adverse cardiovascular events and all-cause mortality when compared with men, even though the risk of major adverse limb events was the same between sexes over a 30-month follow-up period.^42^ Other cohort studies report lower rates of PAD diagnosis, complications, and intervention in women.^43^ Understanding the cause of these disparities is critical in surgical decision-making and treatment outcomes.

## Factors contributing to gender inequalities in PAD

Applying the WHO framework for gender evaluation of healthcare equity, we evaluated potential contributing factors for gendered differences in PAD. By using this social constructivist approach, we sought to explain the key underlying biological, clinical, and societal contributory mechanisms for the PAD-related gender inequalities reported above.

### Biological factors

Sex-related changes in disease biology and pathophysiology at a cellular, hormonal, and physiological level result in differences of disease presentation, progression, and responses to treatment. *Table 2* summarizes the key biological variables that contribute to gender inequality in women.

**Table 2:** Key biological variables that contribute to gender inequality in women with peripheral artery disease (PAD)

Biological	
Female vs. male biology	• Higher risk of thrombosis.^48^• Smaller vessel size.^35^• Unclear impact of antithrombotic therapy and bleeding events.^24,25^
Hormones	• Pregnancy and pre-eclampsia independently predict acute peripheral arterial events.^57^• Higher rates associated with maternal placental syndrome.^55^• Increased cardiovascular mortality and hospitalization for PAD associated with maternal/foetal complications.^56^• Higher rates associated with use of oral contraceptives.^51^• Conflicting impact on PAD risk from hormone replacement therapy.^52–54^
Comorbid illness and cardiovascular risk	• Higher PAD risk with comorbidities, e.g. hypertension, diabetes mellitus and chronic kidney disease.^9^• Associated with depression cluster and higher rates of amputation.^62,63^

#### Differences between female and male biology

PAD is caused primarily by atherosclerosis and thrombosis. How sex impacts PAD pathogenesis is unclear; however, genetic and epigenetic factors,^44^ arterial structure, function, and health,^45^ and differences in response to environmental, physical and/or humoral stresses, and ageing^46^ can impact atherosclerosis progression and pathobiology. Interestingly, a recent study reported that 66% of large peripheral arteries examined in patients with CLTI were blocked by thrombus, in the absence of significant atherosclerosis.^47^ Women have a higher platelet count, and platelets from females have a higher reactivity when compared with men.^48^ It is tantalizing to speculate that sex differences play a role in the risk of thrombosis in PAD; however, further evidence is needed to confirm this. Interestingly, antithrombotic therapy for PAD was associated with higher bleeding complications in women, which may influence prescribing practices,^24^ but the recent COMPASS trial showed comparable bleeding rates between sexes in PAD^25^, highlighting the need to understand sex-dependent pharmacokinetics. Women also have more target lesions in smaller vessels with greater diameter stenosis, length, and multilevel disease.^35^ It is also important not to discount microvascular dysfunction as a mechanism for sex-dependent differences.^45^ Because the majority of pre-clinical studies examining PAD use male animals,^49^ further research efforts using pre-clinical and patient studies in both sexes are essential to increase our understanding of sex-dependent pathophysiology impacting prevalence, clinical manifestations, and outcomes to treatment.

#### Influence of hormones and pregnancy

Reproductive and hormonal factors can influence cardiovascular disease later in life.^50^ In some observational studies, women taking oral contraceptives had higher rates of PAD,^51^ and hormone replacement therapy (HRT) showed conflicting effects.^52–54^ A 3.8-fold increase in PAD risk was associated with maternal placental syndrome.^55^ In a large cohort study, maternal and foetal complications were associated with increased cardiovascular mortality and increased hospitalization for PAD.^56^ Pregnancy and pre-eclampsia were also independent predictors of acute peripheral arterial events,^57^ highlighting the importance of pregnancy and foetal complications as a risk factor for PAD.

#### Differing comorbid illness and risk factors

Major risk factors for PAD include hypertension, raised serum cholesterol, diabetes mellitus, chronic kidney disease, and cigarette smoking. Women with PAD have a greater association with these comorbidities than women without PAD.^9^ It is generally more common for men with IC to smoke;^58^ however, women who smoke have an equal or greater PAD risk, including from second-hand smoke.^59^ Worse outcomes may also stem from different perceptions of limb loss. For example, amputation can affect body image,^60^ cognitive function,^61^ and psychosocial adjustment,^60^ and depression is more prevalent in women than men with PAD, associating with worse health status.^62,63^

### Clinical factors

How individuals engage with healthcare services, their relationships with treating clinicians and how our health systems research, diagnose, and treat PAD are important clinical factors influencing gendered differences. These social constructs can enhance or detract from PAD awareness, equitable management, and the quality of evidence that the treatment of PAD is based on women. *Table 3* describes the health system factors contributing to gendered differences in PAD treatment.

**Table 3:** Key clinical and health system variables that contribute to gender inequality in women with peripheral artery disease (PAD)

Clinical	
Healthcare provider awareness	• Bias of male predominance in PAD.^10^• Low awareness of female PAD.^64^• Misdiagnoses.^14^
Low health literacy and PAD awareness	• Poor awareness of PAD risk and preventative strategies^64,65^ amplified by intersectionality.^69^• Minimize symptoms; less likely to discuss with practioners.^66,68^
Inadequate diagnostic criteria	• Lower ankle-brachial index (ABI) ratios than men.^70^• ABI ratio as a measure of functional impairment inconsistent between sexes.^71^• Discrepancy in self-screening questionnaires.^72^
Evidence paucity	• Underrepresentation in clinical trials (*Figure 2*).• Lack of standardization in gender-related differences reported in clinical research.^73^

#### Healthcare provider awareness

Disease literacy and awareness of PAD risks are poor amongst women and healthcare providers, and healthcare providers are less likely to recognize PAD in women compared with men.^64^ Although women consult general practitioners at similar rates to men prior to their diagnosis with PAD, they are more likely to be misdiagnosed with other conditions, including musculoskeletal disorders.^14^ Unfamiliarity and a lack of knowledge could contribute to under-recognized symptoms and mismanagement of disease.

In a cross-sectional, population-based telephone survey, knowledge gaps of PAD were most evident in those at highest risk. Women had a greater knowledge base than men, but only 14% of women were aware that PAD increased the risk of MI or death.^65^ Furthermore, women were less likely to discuss PAD with their treating clinicians.^66^ This is not surprising since a bias towards an underestimation of pain in women exists^67^ and women may minimize their symptoms and underestimate risks.^68^ Intersectionality contributes to this further; US Hispanic and non-Hispanic black women were less aware of PAD than white women.^69^

#### Diagnostic criteria

Although there are reports that healthy women have a lower ABI than men,^70^ the association of lower ABIs with functional impairment in PAD is inconsistent between the sexes.^71^ This may be due to multiple factors, including calf-muscle size, which could influence occlusion pressures or that women present with atypical disease and are less likely to align with clinical staging criteria.^20^ There is also a significant discrepancy between the PAD symptoms women report on self-screening questionnaires and the symptoms scored by treating doctors, but less so for men,^72^ highlighting differences in perception between the sexes.

#### Evidence paucity

**Figure 2:**
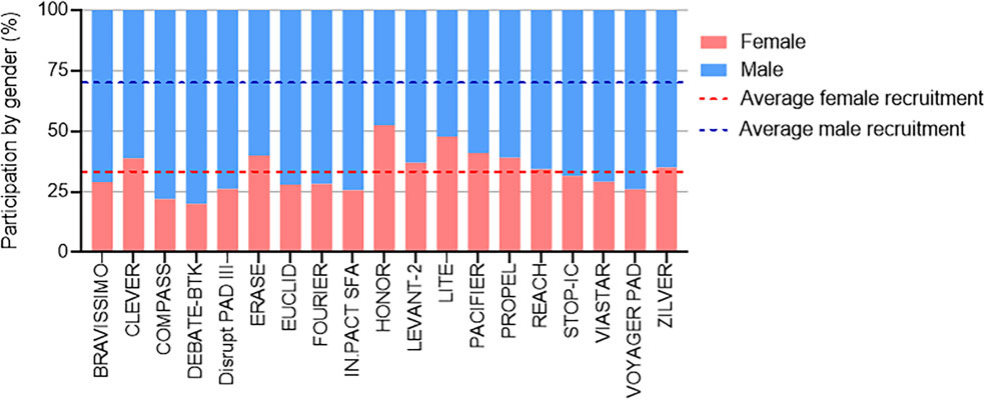
Sex representation in peripheral artery disease-related clinical trials from 2011–2021.

Publication of data that lacks gender, racial, and ethnic diversity can result in clinical care that is not applicable and may be less effective or even harmful to identified groups of the population. Women represent only ∼33% of study participants in trials of intervention for PAD in the last 10 years (*Figure 2*). Poor representation of women in clinical trials is multifactorial and complex to address. For PAD clinical trials, poor enrolment of women may be exacerbated by inclusion criteria, but also by social and clinical factors elaborated on in this review. To improve gender diversity in clinical research, Steinberg *et al*. propose a standardized system of reporting sex-dependent changes in pathogenesis, prognosis, or treatment outcome, including those individuals who are transgender or non-binary.^73^

### Societal factors

The social structures that women live, work, and relate to are important system factors that contribute to overall gender inequality but can also impact gendered differences in PAD. These include the socioeconomic determinants of disease, the value and role of women in society, and geographical patterns associated with disease (*Table 4*). Direct causal evidence for social drivers of inequality is limited. In this section, we present associations with social factors that drive inequality in health in general, applying it to what is known about PAD.

**Table 4:** Key societal variables that may contribute to gender inequality in women with peripheral artery disease (PAD)

Societal	
Socioeconomic status	• Lower socioeconomic status than men globally.^74^• Higher prevalence from low and middle-income countries.^3^
Geographical factors	• Impact of climate/environment.^77,78^• Physical and cultural barriers reducing access to care.^79^
Gendered roles	• Greater domestic demands result in less engagement with doctors for preventative care.^80^• More accepting of reduced exercise tolerance, pain, and functional restrictions.^84,85^• Diagnosis restricts lifestyle and quality of life.^85–87^• Less likely to receive treatment.^85–87^
Access to resources	• Reduced health insurance, hospital care and physician visits^81^; patients without health insurance more likely to receive amputation than revascularization.^83^• Lower education attainment contributes to increased PAD prevalence^75^ and a 2-fold increase in PAD-associated hospitalization risk.^76^
Clinician diversity	• Improved patient outcomes with gender concordance.^88–90^• Improved patient outcomes when treated by female clinicians.^89,92,93^• Women underrepresented in leadership roles.^96^• Women underrepresented in PAD guidelines.^13,18–20^

#### Socioeconomic status

Women have a lower socioeconomic standing than men in most nations, attributed to income inequalities, levels of education, value of women, carer responsibilities, and quality of life.^74^ The incidence of PAD is greater in LMIC, rising most rapidly in women.^3^ Since lower socioeconomic status is associated with increased PAD prevalence and PAD-associated hospitalization risk,^75,76^ the higher poverty and socioeconomic disparities experienced by women globally may contribute to increased rates of PAD in women.

#### Geographical factors

Women in LMIC are impacted by access and environmental issues, which impact their PAD risk and outcomes. Air pollution is a significant environmental risk factor for PAD.^77^ Women, who are disproportionately affected by indoor pollution, passive smoke, and poor sanitation, may have a higher overall cardiovascular mortality as a result,^78^ although the impact on PAD is unclear. Physical access to healthcare providers in LMIC presents additional challenges for women, where care may be delayed if the patient is unaccompanied by a male or where they may be refused access to public transport because of their sex.^79^ These barriers are amplified when intersecting with other sociocultural factors, including reduced financial resources and access to education. Collectively, these may contribute to gender disparities observed in PAD outcomes.

#### Gender roles within society and access to healthcare

Greater domestic demands contribute to women having less engagement with preventative care for cardiovascular diseases.^80^ Despite higher health needs, women have less hospital care, reduced clinician visits, and lower levels of health insurance and economic resources than men in general.^81^ In LMIC, these healthcare access barriers are amplified.^82^ Thus, economic factors may significantly influence how effectively women with PAD can access care. For example, health insurance increases the likelihood of receiving limb-saving revascularization vs. amputation.^83^

Compared with men with similar PAD disease stages, women report significantly more functional limitations, pain, and mood disturbances than men.^84^ Qualitative studies show that women with PAD are more accepting of reductions in exercise tolerance and pain, and experience considerable functional restrictions compared with women without PAD, even with very mild disease.^85^ In women <50 years old, IC significantly impacted health-related quality of life with severe limitations on the physical requirements of daily living and employment.^86^ Older women, even with mild PAD, are more isolated and less likely to leave their immediate neighbourhood compared with other ambulatory women in the community.^85^ In qualitative studies, women tend to describe PAD symptoms more in terms of functional limitations and disability than men.^87^

#### Lack of diversity in treating clinicians

Gender and ethnic diversity in clinical teams improves performance, resulting in superior patient outcomes.^88^ Clinician diversity improves clinical care by enhancing patient satisfaction and trust in patient-clinician relationships, and through improving access and utilization of healthcare services, and reducing post-operative deaths, readmissions, or major complications.^89,90^ A total of 14% of vascular surgeons and trainees in the United Kingdom are women.^91^ A lack of female physicians may contribute to the higher adverse outcomes seen in women with cardiovascular diseases, including PAD.^89,92,93^ Gender discordance between the surgeon and patient could negatively affect the relationship between the physician and patient.^89^ Vascular training programmes are currently directed to address gender imbalances.^94^ More research is needed to understand physician-patient relationships, unconscious and conscious biases, and their impact on PAD healthcare outcomes.

The diversity and inclusion in the authorship, peer review, and editorial processes have an impact on publication biases. Although female academic vascular surgeons in the US hold more NIH grants, they have less publications and citations than male vascular surgeons.^95^ Women also continue to be underrepresented in senior academic leadership roles in vascular surgery and in editorial processes, especially Black, Indigenous, or women of colour.^96^ In the four recent PAD guidelines, women comprised ∼7%,^13^ ∼35%,^20^ ∼38%, and ∼28% of the authorship team. Consequently, research, publications, and policies related to PAD may not be fully reflective of gendered perspectives.

## Knowledge gaps and future research

This review evaluates how interacting gender-based inequalities impact the care and prognosis of women with PAD from a social constructivist perspective. Whilst this approach facilitated a broader discussion on the contributors to PAD gender inequalities, it has also highlighted limited evidence for specific causal associations for PAD outcomes. There are still major gaps in knowledge: from public and health practitioner awareness of PAD to a lack of treatment outcomes for women with PAD because of a lack of recruitment of clinical trials, to our understanding of pathogenesis that is sex dependent. A greater understanding of how the positioning of women in society impacts PAD health outcomes is needed, as well as research into biological sex differences.

This paper has focused on gender-related differences, but we recognize the role that intersectionality also plays in health inequalities. In line with our social constructivist approach, where possible, we have touched upon how race, socioeconomic status, and sexuality are also important markers of diversity, but it is beyond the scope of this paper to expand in detail into these important intersectional differences. Our review is also limited by publication bias and the paucity of evidence from LMICs on gender-related disparities. Steps to address gender-bias in research have been taken through, such as the NIH guidelines on the inclusion of women and minorities as subjects in clinical research^97^ and the Gender Equality in Academia and Research tool.^98^ Widespread implementation of these policies in all aspects of PAD research is needed.

## Data Availability

No new data were generated or analysed in support of this research.
